# Nucleated red blood cells are a late biomarker in predicting intensive care unit mortality in patients with COVID-19 acute respiratory distress syndrome: an observational cohort study

**DOI:** 10.3389/fimmu.2024.1313977

**Published:** 2024-01-18

**Authors:** Götz Schmidt, Arnd Martens, Christian Koch, Melanie Markmann, Emmanuel Schneck, Ulrich Matt, Matthias Hecker, Khodr Tello, Matthias Wolff, Michael Sander, István Vadász

**Affiliations:** ^1^ Department of Anesthesiology, Intensive Care Medicine and Pain Therapy, Justus Liebig University Giessen, Giessen, Germany; ^2^ Department of Internal Medicine V, Universities of Giessen and Marburg Lung Center (UGMLC), Excellence Cluster Cardiopulmonary Institute (CPI), Member of the German Center for Lung Research (DZL), Justus-Liebig University, Giessen, Germany; ^3^ Department of Internal Medicine II, Universities of Giessen and Marburg Lung Center (UGMLC), Excellence Cluster Cardiopulmonary Institute (CPI), Member of the German Center for Lung Research (DZL), Justus-Liebig University, Giessen, Germany

**Keywords:** normoblast, NRBC, SARS-CoV-2, pandemic, ARDS, disease severity score

## Abstract

**Background:**

Nucleated red blood cells (nRBC) are precursor cells of the erythropoiesis that are absent from the peripheral blood under physiological conditions. Their presence is associated with adverse outcomes in critically ill patients. This study aimed to evaluate the predictive value of nRBC on mortality in intensive care unit (ICU) patients with COVID-19 acute respiratory distress syndrome (ARDS).

**Material and methods:**

This retrospective, observational cohort study analyzed data on 206 ICU patients diagnosed with COVID-19 ARDS between March 2020 and March 2022. The primary endpoint was ICU mortality, and secondary endpoints included ICU and hospital stay lengths, ventilation hours, and the time courses of disease severity scores and clinical and laboratory parameters.

**Results:**

Among the included patients, 68.9% tested positive for nRBC at least once during their ICU stay. A maximum nRBC of 105 µl^-1^ had the highest accuracy in predicting ICU mortality (area under the curve of the receiver operating characteristic [AUCROC] 0.780, *p* < 0.001, sensitivity 69.0%, specificity 75.5%). Mortality was significantly higher among patients with nRBC >105 µl^-1^ than ≤105 µl^-1^ (86.5% vs. 51.3%, *p* = 0.008). Compared to patients negative for nRBC in their peripheral blood, those positive for nRBC required longer mechanical ventilation (127 [44 - 289] h vs. 517 [255 - 950] h, *p* < 0.001), ICU stays (12 [8 – 19] vs. 27 [13 – 51] d, *p* < 0.001), and hospital stays (19 [12 - 29] d vs. 31 [16 - 58] d, *p* < 0.001). Peak Sepsis-related Organ Failure Assessment (SOFA), Simplified Acute Physiology Score, P_a_O_2_/F_i_O_2_, interleukin-6, and procalcitonin values were reached before the peak nRBC level. However, the predictive performance of the SOFA (AUCROC 0.842, *p* < 0.001) was considerably improved when a maximum SOFA score >8 and nRBC >105 µl^-1^ were combined.

**Discussion:**

nRBC predict ICU mortality and indicate disease severity among patients with COVID-19 ARDS, and they should be considered a clinical alarm signal for a worse outcome. nRBC are a late predictor of ICU mortality compared to other established clinical scoring systems and laboratory parameters but improve the prediction accuracy when combined with the SOFA score.

## 1 Introduction

The global spread of the novel severe acute respiratory syndrome coronavirus 2 (SARS-CoV-2) has led to the widespread occurrence of coronavirus disease 2019 (COVID-19) since 2020, which, at worst, presents with acute respiratory distress syndrome (ARDS) and multi-organ dysfunction or failure, including the heart, kidney, liver, and brain (1). Due to simultaneous infections of large populations worldwide, the burden of patients requiring extensive treatment in intensive care units (ICU), including the need for mechanical ventilation and extracorporeal membrane oxygenation (ECMO), led to regional overload of ICU capacities.

The COVID-19 pandemic became a global health crisis, with over 769 million infections and 6.9 million deaths (2). Since free ICU capacities were initially limited, early discussions already focused on patient selection and triage. Therefore, various parameters have been studied to predict adverse outcomes following COVID-19-associated ARDS (3–5). While ICU capacities are currently preserved due to a generally growing immunity, vaccination, and virus evolution, which all reduce disease severity, risk prediction triggering specific therapies in patients with high risk for severe COVID-19, including ARDS, remains vital (6).

Among the diverse parameters studied, hematological parameters, such as neutrophils, lymphocytes, and the neutrophil-lymphocyte ratio, coagulation biomarkers, and proinflammatory cytokines could predict disease severity and poor outcomes (5, 7–9). However, nucleated red blood cells (nRBC), which are precursor cells of the erythropoiesis, have not been studied in detail for their predictive value among ICU patients with COVID-19. nRBC are not present in the peripheral blood in healthy patients under physiological conditions. However, they can appear in the peripheral blood during critical illness. Therefore, the occurrence and amount of nRBC observed in the peripheral blood could predict mortality and disease severity in medical and surgical ICU patients during sepsis and non-COVID-19-associated ARDS (10–15).

The pathophysiological mechanisms that flush nRBC from the bone marrow into the peripheral blood remain incompletely understood. Nevertheless, the release of nRBC was found to be associated with inflammatory parameters, such as interleukin (IL)-3, IL-6, and hypoxemia, with higher erythropoietin levels and lower partial pressures of oxygen (P_a_O_2_) observed before nRBC appeared (16, 17). Therefore, nRBC release is considered an indicator of critically ill patients with high mortality risk, indicating disturbed bone marrow function triggered by inflammation and hypoxemia.

Since ARDS is triggered by the SARS-CoV-2-induced cytokine storm, ultimately resulting in hypoxemia in patients requiring ICU therapy, both mechanisms known to trigger nRBC release are present in patients with COVID-19 (18). Furthermore, since COVID-19 is considered a multi-system disease, SARS-CoV-2 has been shown to affect the bone marrow (19, 20). Therefore, the extent to which nRBC can predict mortality among patients with COVID-19 ARDS requiring ICU treatment remains unknown. and predictivity could be impaired by SARS-CoV-2 affected bone narrow function. Nonetheless, dysregulation of hematopoiesis was identified as a marker for severe COVID-19 (21). Therefore, this study aimed to evaluate the frequency of nRBC occurrence and the predictive value of different values of nRBC count in patients with COVID-19 ARDS. Moreover, other predictive measurements, such as standard disease severity scores and clinical parameters, along with their peak values, were analyzed in comparison to the timepoint of nRBC occurrence, and the potential improvement in predicting ICU mortality through the combination of disease severity scores and nRBC was assessed.

## 2 Materials and methods

### 2.1 Study design

This retrospective, single-center observational cohort study was approved by the local ethics committee of the medical faculty of Justus Liebig University, Giessen, Germany (AZ 36/22). It retrospectively screened the clinical data records of patients who tested positive for or were suspected of having SARS-CoV-2 and required ICU treatment at the tertiary care University Hospital Giessen between March 2020 and March 2022. The patients were admitted to the ICU from the Emergency Department or a general ward or were transferred from other hospitals of a lower care level that were not specialized in the ICU treatment of patients with ARDS, including ECMO.

The inclusion criteria were age ≥18 years, ICU stay length ≥24 hours, and confirmed COVID-19. Patients who did not meet ARDS criteria according to the Berlin definition and those who tested positive for SARS-CoV-2 but required ICU treatment for other reasons, such as trauma and postoperative care, were excluded. Further exclusion criteria were ICU stay <24 hours, refusal of life-sustaining therapies at ICU admission, such as intubation, invasive ventilation, and cardiopulmonary resuscitation. Patients with known hematological neoplasia and cyanotic heart defects were excluded because both conditions could potentially induce and interact with nRBC release.

Sufficient availability of nRBC measurements was defined as at least one measurement every two days. Consequently, patients with insufficient availability of nRBC measurements and clinical information were excluded from the analysis. ICU treatment was provided to all patients according to the available national and international guidelines and recommendations for treating ARDS and COVID-19 ARDS at that time (22, 23). Because the data were anonymized, the requirement to obtain informed consent was waived due to the study’s retrospective design, which only used data from clinical and administrative records.

### 2.2 Data acquisition

After eligible patients were identified, clinical data records and diagnostic results were reviewed manually. All data were extracted from the electronic automated patient data management system (IMESO^®^ GmbH, Giessen, Germany). Baseline characteristics were registered at ICU admission and included age, sex, body mass index, cardiovascular risk factors, and relevant comorbidities such as peripheral artery disease, carotid artery stenosis, preexisting pulmonary disease, pulmonary hypertension, chronic kidney disease, and chronic immunosuppression. Further parameters concerning the SARS-CoV-2 variant and vaccination status, classified as unvaccinated, vaccinated, and vaccinated with an additional booster dose, were assessed at ICU admission. ARDS severity according to the Berlin definition, the need for non-invasive or invasive ventilation, prone positioning and ECMO therapy, and implementation of specific pharmacologic strategies against COVID-19, such as dexamethasone, IL-6 antagonists, or JAK inhibitors, were recorded at admission and during the ICU stay (24). Ventilation parameters were analyzed at admission and during ICU stay, including the fraction of inspired oxygen (F_i_O_2_), positive end-expiratory pressure (PEEP), peak inspiratory pressure, compliance, and breathing rate. Laboratory and blood gas measurements were recorded throughout the ICU stay, including the arterial partial pressure of carbon dioxide (P_a_CO_2_), P_a_O_2_, P_a_O_2_/F_i_O_2_ ratio, serum procalcitonin (PCT), IL-6, and lactate dehydrogenase.

Clinical disease severity scores, such as the Sepsis-related Organ Failure Assessment (SOFA), the Acute Physiology and Chronic Health Evaluation (APACHE) II, and Simplified Acute Physiology Score (SAPS) II, were calculated at ICU admission and daily during the ICU stay. To characterize overall disease severity at admission and during the ICU treatment, peak values of the above-mentioned scores, ARDS severity, laboratory parameters, and ECMO usage were reported. Furthermore, ICU and hospital stay lengths and mechanical ventilation duration were derived from administrative records. nRBC were measured within the blood count in the routine clinical laboratories during the ICU stay. Every nRBC measurement was registered during the ICU stay, and peak values were reported. Clinical outcome data were classified as death or survivor discharged from ICU to rehabilitation, a general ward, or home.

### 2.3 Endpoints

The primary endpoint was ICU mortality. The secondary endpoints were the discharge rate, ICU and hospital stay lengths, and ventilation duration.

### 2.4 Statistical analysis

Categorical data are presented as numbers and percentages and were compared between groups using Chi-squared or Fisher’s exact tests. Continuous variables are presented as medians and interquartile ranges (IQR) and were compared between groups using the Mann–Whitney–Wilcoxon test. ICU mortality was initially stratified by maximum nRBC count. The area under the curve of the receiver operating characteristic (AUCROC) was subsequently calculated to determine the discriminatory power of peak nRBC, nRBC at admission, and disease severity scores in predicting the primary endpoint. Optimal discriminatory levels were identified according to Youden’s index. Outcomes were compared between patients above and below the calculated cut-offs. Event rates were calculated using the Kaplan-Meier method, while statistical differences were assessed using the log-rank test. Cox regression models were used to analyze the hazard ratios for ICU mortality stratified by the nRBC count. When the predictive value of nRBC was compared with other clinical parameters and scores, AUCROC were compared using a z-test. Groups stratified by the calculated nRBC cut-off or by survivors and non-survivors were created, and inter-group differences in disease severity scores and other clinical parameters over time were evaluated using two-way analysis of variance for repeated measurements and Tukey’s *post-hoc* test. A two-tailed p-value <0.05 was generally considered statistically significant. Statistical analyses were performed using IBM SPSS Statistics (version 28.0.0.1; IBM, Armonk, NY, USA).

## 3 Results

**Figure 1:**
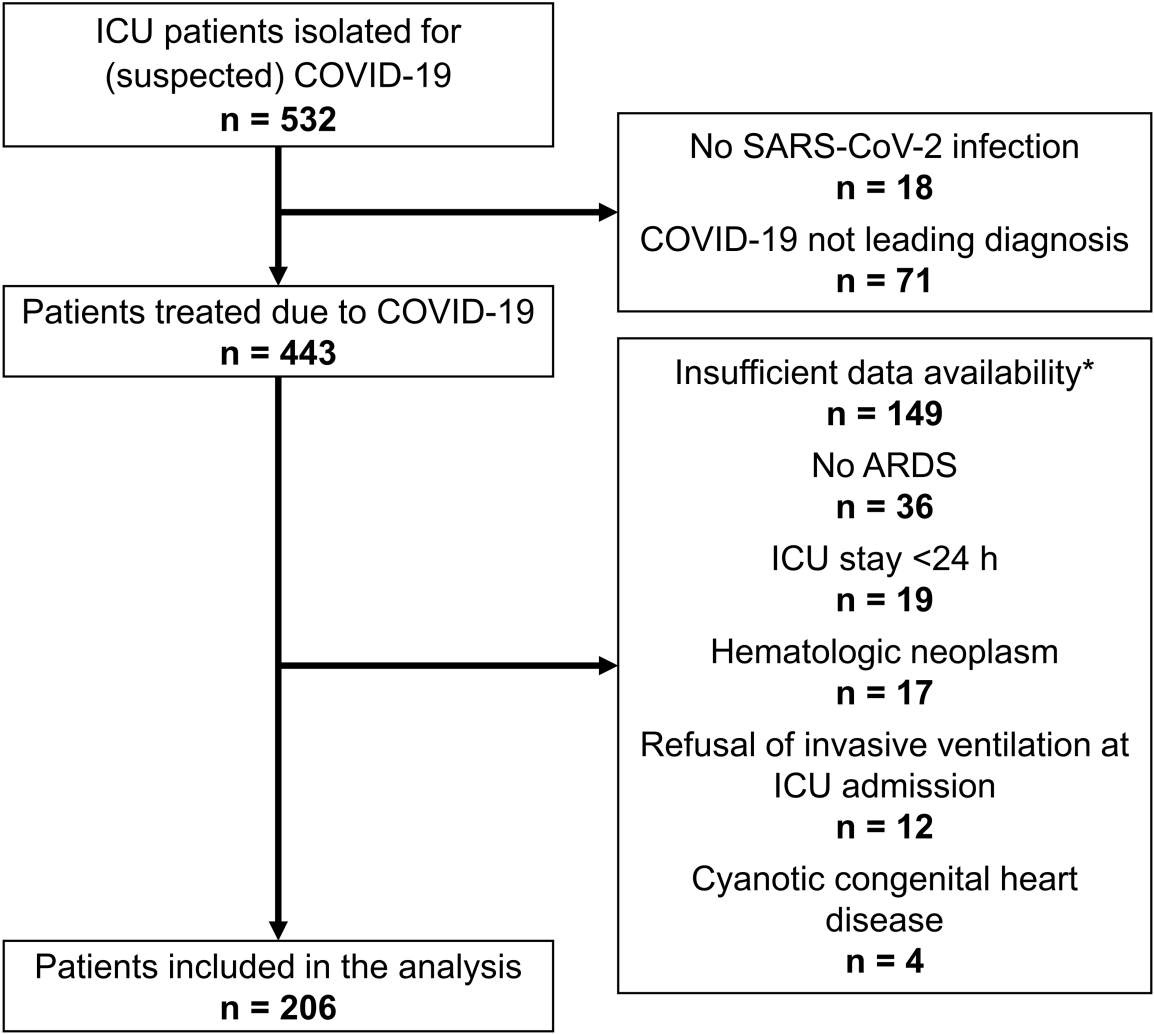
Study flowchart. *Sufficient availability of nRBC measurements was defined as at least one measurement every two days.

Five hundred thirty-two patients were initially retrospectively screened, and 206 were included in the final analysis. The study flowchart is shown in **Figure 1**. Most analyzed patients (142, 68.9%) were measured positive for nRBC in the peripheral blood during their ICU stay.

### 3.1 Patient characteristics

The patients’ characteristics are shown in **Table 1**. No differences in biometrics and clinical history were observed between nRBC-positive and nRBC-negative patients. Patients admitted from a lower care level hospital were more frequently measured positive for nRBC during their ICU stay, and blasts were more frequently found in patients who measured positive for nRBC. Vaccination status, SARS-CoV-2 specific medical therapy, and SARS-CoV-2 variants did not differ between nRBC-positive and nRBC-negative patients. However, patients who measured positive for nRBC during ICU treatment had significantly greater disease severity, indicated by less mild and moderate but more severe ARDS, higher maximum disease severity scores, higher maximal IL-6 and PCT levels, lower minimal P_a_O_2_/F_i_O_2_, greater need for mechanical non-invasive or invasive ventilation, and greater need for ECMO therapy.

**Table 1:** Patient characteristics.

	total (n = 206)	nRBC negative (n = 64)	nRBC positive (n = 142)	*p*
Biometrics				
age [IQR] - years	65 [55 - 72]	64 [54 - 69]	66 [57 - 74]	0.107
body mass index [IQR] - kg/m^2^	27.8 [24.7 - 31.9]	28.6 [24.7 - 33.6]	27.8 [24.7 - 31.2]	0.545
male - % (no.)	68.0 (140)	70.3 (45)	66.9 (95)	0.746
Clinical history				
arterial hypertension - % (no.)	66.0 (136)	64.1 (41)	66.9 (95)	0.811
diabetes mellitus- % (no.)	37.9 (78)	39.1 (25)	37.3 (53)	0.934
active smoker - % (no.)	5.3 (11)	4.7 (3)	5.6 (8)	1.000
ex-smoker - % (no.)	14.1 (29)	12.5 (8)	14.8 (21)	0.825
coronary artery disease - % (no.)	22.8 (47)	23.4 (15)	22.5 (32)	1.000
prior myocardial infarction - % (no.)	9.2 (19)	14.1 (9)	7.0 (10)	0.177
peripheral artery disease - % (no.)	7.3 (15)	4.7 (3)	8.5 (12)	0.401
carotid stenosis - % (no.)	3.9 (8)	6.3 (4)	2.8 (4)	0.258
chronic obstructive pulmonary disease - % (no.)	10.2 (21)	14.1 (9)	8.5 (12)	0.326
bronchial asthma - % (no.)	8.3 (17)	9.4 (6)	7.7 (11)	0.905
pulmonary fibrosis - % (no.)	3.4 (7)	1.6 (1)	4.2 (6)	0.439
pulmonary hypertension - % (no.)	3.4 (7)	4.7 (3)	2.8 (4)	0.679
chronic kidney disease - % (no.)	19.4 (40)	12.5 (8)	22.5 (32)	0.135
chronic immunosuppression - % (no.)	4.4 (9)	1.6 (1)	5.6 (8)	0.279
pregnant - % (no.)	0.5 (1)	0.0 (0)	0.7 (1)	1.000
transfer from lower level of care - % (no.)	43.2 (89)	29.7 (19)	49.3 (70)	**0.013**
Progenitor cells in peripheral blood				
maximum nRBC [IQR] - n/µl	75 [0 - 643]	0 [0 - 0]	265 [70 - 1473]	**<0.001**
number of nRBC proofs per patient (IQR) - no.	3.0 [0.0-10.0]	0.0 [0.0-0.0]	6.5 [2.8-14.0]	**<0.001**
blasts - % (no.)	22.8 (47)	9.4 (6)	28.9 (41)	**0.004**
Virus variants				
wildtype - % (no.)	77.2 (159)	71.9 (46)	79.6 (113)	0.298
Alpha - % (no.)	12.1 (25)	18.8 (12)	9.2 (13)	0.085
Delta - % (no.)	9.7 (20)	9.4 (6)	9.9 (14)	1.000
Omicron - % (no.)	1.5 (3)	0.0 (0)	2.1 (3)	0.554
Vaccination status				
unvaccinated - % (no.)	93.2 (192)	89.1 (57)	95.1 (135)	0.137
vaccinated - % (no.)	4.4 (9)	4.7 (3)	4.2 (6)	1.000
booster shot - % (no.)	0.5 (1)	1.6 (1)	0.0 (0)	0.311
SARS-CoV-2 specific medical therapy				
Dexamethasone - % (no.)	88.8 (183)	85.9 (55)	90.1 (128)	0.517
IL-6 antagonists - % (no.)	6.3 (13)	6.3 (4)	6.3 (9)	1.000
JAK-inhibitors - % (no.)	1.0 (2)	0.0 (0)	1.4 (2)	1.000
Maximum Berlin classification				
mild - % (no.)	2.9 (6)	7.8 (5)	0.7 (1)	0.012
moderate - % (no.)	34.5 (71)	57.8 (37)	23.9 (34)	<0.001
severe - % (no.)	62.6 (129)	34.4 (22)	75.4 (107)	<0.001
Maximum scores				
SOFA max [IQR] - no.	8 [6 - 10]	5 [4 - 7]	9 [8 - 11]	<0.001
APACHE II max [IQR] - no.	26 [21 - 30]	21 [18 - 26]	27 [13 - 31]	<0.001
SAPS II max [IQR] - no.	55 [44 - 63]	43 [36 - 53]	59 [51 - 65]	<0.001
Laboratory parameters				
maximum interleukin 6^1^ [IQR] - pg/ml	208 [70 - 804]	117 [37 - 325]	334 [118 - 1276]	<0.001
minimum P_a_O_2_/F_i_O_2_ [IQR] - mmHg	83 [61 - 117]	115 [83 - 153]	70 [56 - 97]	<0.001
maximum procalcitonin^2^ [IQR] - µg/ml	6 [4 - 15]	5 [2 - 6]	8 [5 - 18]	<0.001
Mechanical Ventilation				
non-invasive - % (no.)	65.0 (134)	84.4 (54)	56.3 (80)	**<0.001**
invasive - % (no.)	72.3 (149)	37.5 (24)	88.0 (125)	**<0.001**
Extracorporeal membrane oxygenation				
ECMO initiation - % (no.)	25.2 (52)	3.1 (2)	35.2 (50)	**<0.001**
VV-ECMO - % (no.)	24.8 (51)	1.6 (1)	35.2 (50)	**<0.001**
VAV-ECMO - % (no.)	0.5 (1)%	1.6 (1)	0.0 (0)	0.311

APACHE II, Acute Physiology and Chronic Health Evaluation II; ECMO, extracorporeal membrane oxygenation; nRBC, nucleated red blood cells; SAPS II, Simplified Acute Physiology Score II; SOFA, sepsis-related organ failure assessment score; 1. nRBC- = 52, nRBC+ = 115, total = 167; 2. nRBC- = 63, nRBC+ = 141. Bold values indicate statistical significance (= all p < 0.05).

### 3.2 Clinical characteristics when nRBC were found

nRBC were found in 1784 laboratory tests during the ICU stays of the analyzed patients, with 1277 and 507 positive measurements observed in ICU non-survivors and survivors, respectively. The clinical characteristics of patients with positive nRBC measurements are shown in **Table 2**. Non-survivors had higher nRBC counts at later stages in their ICU stay. Mild and moderate ARDS were more frequent in survivors, while severe ARDS was more frequent in non-survivors. Consequently, mechanical ventilation was more often necessary in non-survivors, and non-survivors had worse ventilator parameters, such as F_i_O_2_, PEEP, peak inspiratory pressure, tidal volume, compliance, and breathing rate. Arterial blood gases and laboratory parameters indicated significantly greater disease severity in non-survivors than survivors. Correspondingly, prone positioning and ECMO utilization were significantly greater in non-survivors, and SOFA and SAPS II scores were lower in survivors.

**Table 2:** Clinical characteristics when nucleated red blood cells (nRBC) were found.

	total (n = 1784)	survivor (n = 507)	non- survivor (n = 1277)	*p*
Characteristics				
nRBC count [IQR] - μl^-1^	120 [40 - 410]	60 [30 - 170]	180 [50 - 585]	**<0.001**
intensive care unit day [IQR] - day	18 [9 - 26]	13 [8 - 16]	20 [10 - 39]	**<0.001**
Berlin classification				
none - % (no.)	7.7 (138)	21.1 (107)	2.4 (31)	**<0.001**
mild - % (no.)	12.2 (218)	24.5 (124)	7.4 (94)	**<0.001**
moderate - % (no.)	27 (482)	35.3 (179)	23.7 (303)	**<0.001**
severe - % (no.)	53.0 (946)	19.1 (97)	66.5 (849)	**<0.001**
Ventilation				
mechanical ventilation - % (no.)	97.5 (1740)	92.9 (471)	99.4 (1269)	**<0.001**
non-invasive - % (no.)	3.5 (63)	5.1 (26)	2.9 (37)	**0.031**
invasive - % (no.)	94.0 (1677)	87.8 (445)	96.5 (1232)	**<0.001**
F_i_O_2_^1^ [IQR] - %	65 [45 - 100]	45 [30 - 62]	100 [60 - 100]	**<0.001**
positive end-expiratory pressure^2^ [IQR] - mbar	10 [8 - 12]	9 [7 - 10]	10 [8 - 12]	**<0.001**
peak inspiratory pressure^3^ [IQR] - mbar	26 [23 - 29]	24 [19 - 26]	27 [24 - 30]	**<0.001**
tidal volume^4^ [IQR] - ml	271 [168 - 469]	440 [280 - 540]	230 [148. - 412]	**<0.001**
compliance^4^ [IQR] - ml/mbar	16.4 [9.9-31.9]	32.5 [16.4-45.4]	13.3 [8.8-26.7]	**<0.001**
breathing rate^5^ [IQR] - min^-1^	20 [17 - 25]	20 [17 - 24]	21 [18 - 25]	**0.012**
Prone positioning and extracorporeal membrane oxygenation				
prone positioning - % (no.)	12.8 (228)	9.7 (49)	14.0 (179)	**0.016**
VV-ECMO - % (no.)	61.5 (1098)	32.7 (166)	73.0 (932)	**<0.001**
VAV-ECMO - % (no.)	0.1 (1)	0.0 (0)	0.1 (1)	1.000
Arterial blood gases				
pH^6^ [IQR] - no.	7.40 [7.35 - 7.44]	7.43 [7.39 - 7.46]	7.39 [7.34 - 7.43]	**<0.001**
P_a_CO_2_^6^ [IQR] - mmHg	46 [40 - 51]	43 [38 - 49]	46 [42 - 52]	**<0.001**
P_a_O_2_^6^ [IQR] - mmHg	73 [67 - 82]	76 [70 - 85]	71 [65 - 80]	**<0.001**
P_a_O_2_/F_i_O_2_^7^ [IQR] - mmHg	93 [71 - 167]	174 [117 - 245]	80 [68 - 126]	**<0.001**
Laboratory parameters				
procalcitonin^8^ [IQR] - µg/l	3.7 [1.9 - 6.7]	2.7 [1.4 - 4.3]	4.5 [2.3 - 8.2]	**<0.001**
interleukin 6^9^ [IQR] - pg/ml	59 [24 - 178]	29 [9 - 51]	83 [34 - 263]	**<0.001**
lactate dehydrogenase^10^ [IQR] - U/l	496 [363 - 750]	379 [291 - 516]	540 [414 - 849]	**<0.001**
Scores				
SOFA [IQR]^11^ - no.	7 [4 - 9]	4 [3 - 6]	8 [6 - 9]	**<0.001**
APACHE II^11^ [IQR] - no.	18 [14 - 22]	18 [14 - 22]	18 [14 - 22]	0.214
SAPS II [IQR]^11^ - no.	44 [37 - 51]	39 [36 - 46]	45 [39 - 52]	**<0.001**

n, number of laboratory tests yielding positive results for nRBC. APACHE II, Acute Physiology and Chronic Health Evaluation II; ECMO, extracorporeal membrane oxygenation; FiO2, fraction of inspired oxygen; nRBC, nucleated red cells; P_a_CO_2_: partial pressure of carbon dioxide; P_a_O_2_, partial pressure of oxygen; SAPS II, Simplified Acute Physiology Score II; SOFA, sepsis-related organ failure assessment score; 1. survivors: n = 506, non-survivors: n = 1277, total: n = 1765; 2. survivors: n = 465, non-survivors: n = 1268, total: n = 1733; 3. survivors: n = 468, non-survivors: n = 1269, total: n = 1737; 4. survivors: n = 460, non-survivors: n = 1256, total: n = 1716; 5. survivors: n = 502, non-survivors: n = 1263, total: n = 1765; 6. survivors: n = 489, non-survivors: n = 1272, total: n = 1761; 7. survivors: n = 488, non-survivors: n = 1272, total: n = 1760; 8. survivors: n = 238, non-survivors: n = 488, total: n = 726; 9. survivors: n = 102, non-survivors: n = 223, total: n = 325; 10. survivors: n = 491, non-survivors: n = 1243 total: n = 1734; 11. survivors: n = 506, non-survivors: n = 1213, total: n = 1719. Bold values indicate statistical significance (= all p < 0.05).

### 3.3 nRBC measurements in different subgroups

**Figure 2:**
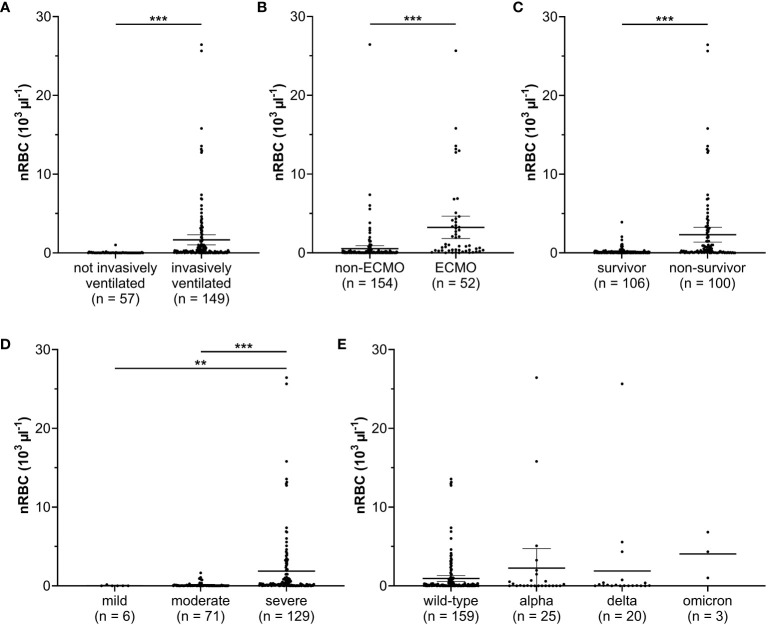
Nucleated red blood cell (nRBC) count was significantly higher in patients who were **(A)** invasively ventilated, **(B)** required extracorporeal membrane oxygenation (ECMO), and **(C)** died during intensive care. **(D)** Patients with moderate or severe acute respiratory distress syndrome had significantly higher nRBC levels during intensive care unit (ICU) stay. **(E)** No significant differences in nRBC counts were observed between the analyzed SARS-CoV-2 variants. **p < 0.01; ***p < 0.001. mean with 95% confidence interval.

The distributions of maximum nRBC counts stratified by different clinical parameters are shown in **Figure 2**. Patients who required invasive ventilation (0 [0 – 20] µl^-1^ vs. 240 [40 – 1080] µl^-1^, *p* < 0.001; **Figure 2A**), ECMO therapy (40 [0 – 165] µl^-1^ vs. 895 [285 – 3805] µl^-1^, *p* < 0.001; **Figure 2B**), and non-survivors (20 [0 – 105] µl^-1^ vs. 355 [55 – 2672] µl^-1^, *p* < 0.001; **Figure 2C**) had significantly higher maximum nRBC counts during their ICU stay. Correspondingly, patients with severe ARDS had higher maximum nRBC levels compared with patients with mild or moderate ARDS (**Figure 2D**). No differences in maximum nRBC counts were observed when stratified by the SARS-CoV-2 variant (wild-type: 80 [0 – 550] µl^-1^, alpha: 30 [0 – 1080] µl^-1^, delta: 45 [0 – 395] µl^-1^; **Figure 2E**).

### 3.4 Outcome

Mortality and survivor rates, mechanical ventilation duration, and ICU and hospital stay lengths are shown in **Table 3**. Patients with a positive nRBC measurement during their ICU stay showed significantly higher mortality, while patients without a positive nRBC measurement were significantly more often discharged from the ICU to a general ward; no differences were observed for discharge to rehabilitation or home. Correspondingly, patients with positive nRBC measurements had significantly longer ventilation durations and ICU and hospital stay lengths.

**Table 3:** Clinical outcomes stratified by nucleated red blood cell positivity.

	total (n = 206)	nRBC negative (n = 64)	nRBC positive (n = 142)	*p*
Outcome				
death - % (no.)	48.5 (100)	20.3 (13)	61.3 (87)	<0.001
survivor - % (no.)	51.5 (106)	79.7 (51)	38.7 (55)	<0.001
discharge to general ward - % (no.)	32.5 (67)	62.5 (40)	19.0 (27)	<0.001
discharge to rehabilitation - % (no.)	16.0 (33)	12.5 (8)	17.6 (25)	0.472
discharge to home - % (no.)	2.9 (6)	4.7 (3)	2.1 (3)	0.377
Length of stays				
duration of mechanical ventilation [IQR] - hours	331 [139 - 780]	127 [44 - 289]	517 [255 - 950]	<0.001
length of intensive care unit stay [IQR] - days	21 [11 - 44]	12 [8 - 19]	27 [13 - 51]	<0.001
length of hospital stay [IQR] - days	24 [11 - 47]	19 [12 - 29]	31 [16 - 58]	<0.001

nRBC, nucleated red blood cells.

**Figure 3:**
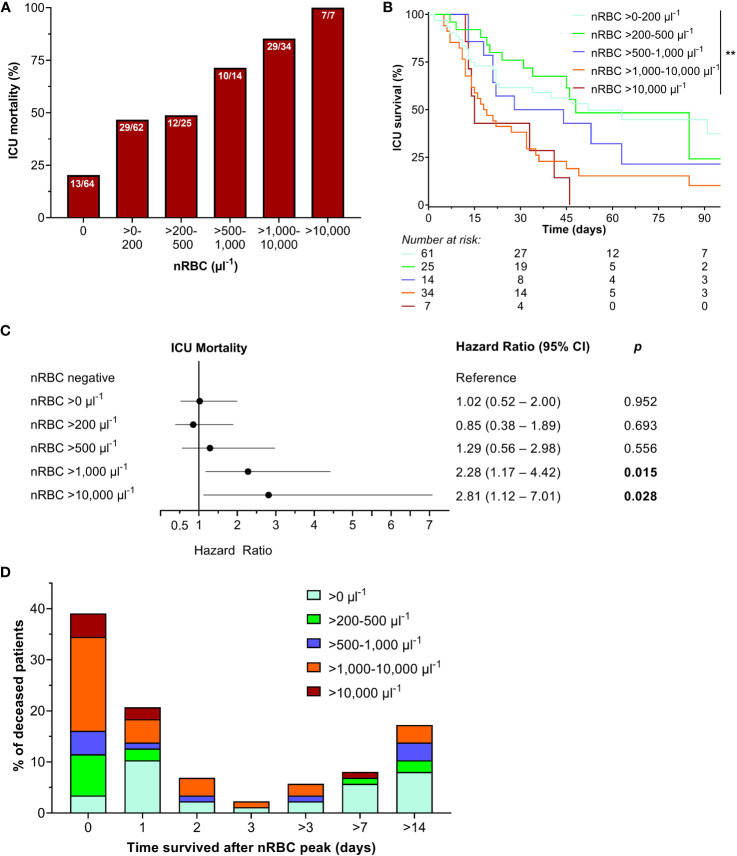
**(A)** Intensive care unit (ICU) mortality increased when stratified by maximum nucleated red blood cell (nRBC) count during the ICU stay, with **(B)** significantly decreasing survival rates with nRBC count. **(C)** Maximum nRBC counts above 1,000 µl^-1^ and 10,000 µl^-1^ were independent risk predictors for ICU mortality. **(D)** Most of the deceased patients died shortly after their individual nRBC peak. **p < 0.01.

When mortality was stratified by maximum nRBC counts, mortality rates increased incrementally from 20.3% (nRBC negative) to 100% (nRBC > 10,000 µl^-1^, **Figure 3A**). Correspondingly, Kaplan-Meier survival rates decreased incrementally with the increase in maximum nRBC count (nRBC >0 µl^-1^: 37.4%, nRBC >200 µl^-1^: 24.2%, >500 µl^-1^: 21.4%, >1,000 µl^-1^: 10.2%, >10.000 µl^-1^: 0.0%, *p* = 0.001; **Figure 3B**). When nRBC negative was set as the reference, nRBC >1,000 µl^-1^ and nRBC >10.000 µl^-1^ were independent risk factors for ICU mortality in the Cox regression model (**Figure 3C**). In addition, 59.8% of the deceased patients with positive nRBC measurements died within 24 hours after their individual nRBC peak, with death occurring the earlier the higher the maximum nRBC count was observed (**Figure 3D**).

**Figure 4:**
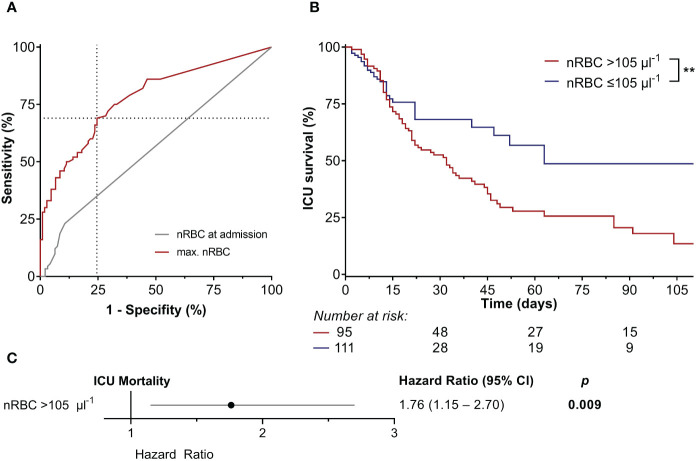
**(A)** The nucleated red blood cell (nRBC) count at intensive care unit (ICU) admission failed to predict ICU mortality. However, a maximum nRBC count of >105 µl^-1^ had the highest accuracy in predicting ICU mortality (area under the curve of the receiver operating characteristic 0.780, *p* < 0.001, sensitivity 69.0%, specificity 75.5%). **(B)** Kaplan-Meier estimates showed significantly higher ICU mortality in patients with a maximum nRBC count >105 µl^-1^, **(C)** which was also an independent risk factor for ICU mortality in the Cox regression model. **p < 0.01.

A maximum nRBC cut-off of >105 µl^-1^ showed the best predictivity for ICU mortality based on the receiver operating characteristic (AUCROC 0.780, *p* < 0.001, Sensitivity 69.0%, Specificity 75.5%; **Figure 4A**). However, a positive nRBC measurement at ICU admission failed to predict ICU mortality (AUCROC 0.560, *p* = 0.159). Correspondingly, survival estimates were significantly higher in patients with maximum nRBC counts ≤105 µl^-1^ than >105 µl^-1^ (48.7% vs. 13.5%, *p* = 0.008; **Figure 4B**), and a maximum nRBC count >105 µl^-1^ was an independent risk factor for ICU mortality in the Cox regression model (**Figure 4C**).

**Figure 5:**
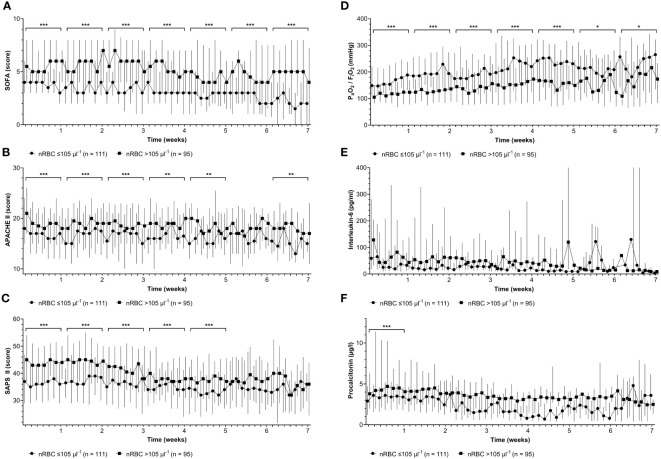
Patients with maximum nucleated red blood cell (nRBC) counts >105 µl^-1^ showed significantly higher **(A)** Sepsis-related Organ Failure Assessment (SOFA) scores, **(B)** higher Acute Physiology and Chronic Health Evaluation (APACHE) II scores, **(C)** higher Simplified Acute Physiology Scores (SAPS) II, and **(D)** lower minimum arterial oxygen partial pressure to fractional inspired oxygen ratios (P_a_O_2_/F_i_O_2_) throughout the intensive care unit stay when compared on a weekly basis (brackets). **(E)** No significant differences were observed for interleukin-6 levels and **(F)** procalcitonin levels later than the first week of the ICU stay. *p < 0.05; **p < 0.01; ***p < 0.001.

The courses of disease severity scores (SOFA, APACHE II, and SAPS II), P_a_O_2_/F_i_O_2_, and IL-6 and PCT levels during the ICU stay are shown in **Figure 5**. Parameters were stratified by and compared between the calculated maximum nRBC count cut-off of 105 µl^-1^ by week. SOFA, APACHE II, and SAPS II scores were significantly elevated throughout the ICU stay (**Figures 5A-C**). P_a_O_2_/F_i_O_2_ was significantly lower in patients with an nRBC count >105 µl^-1^ during the entire ICU treatment (**Figure 5D**). Although no significant differences in IL-6 levels could be detected, patients with nRBC >105 µl^-1^ presented with significantly elevated PCT levels during the first week on ICU (**Figures 5E-F**).

### 3.5 The predictive value of the maximum nRBC count compared to maximum disease severity scores

**Figure 6:**
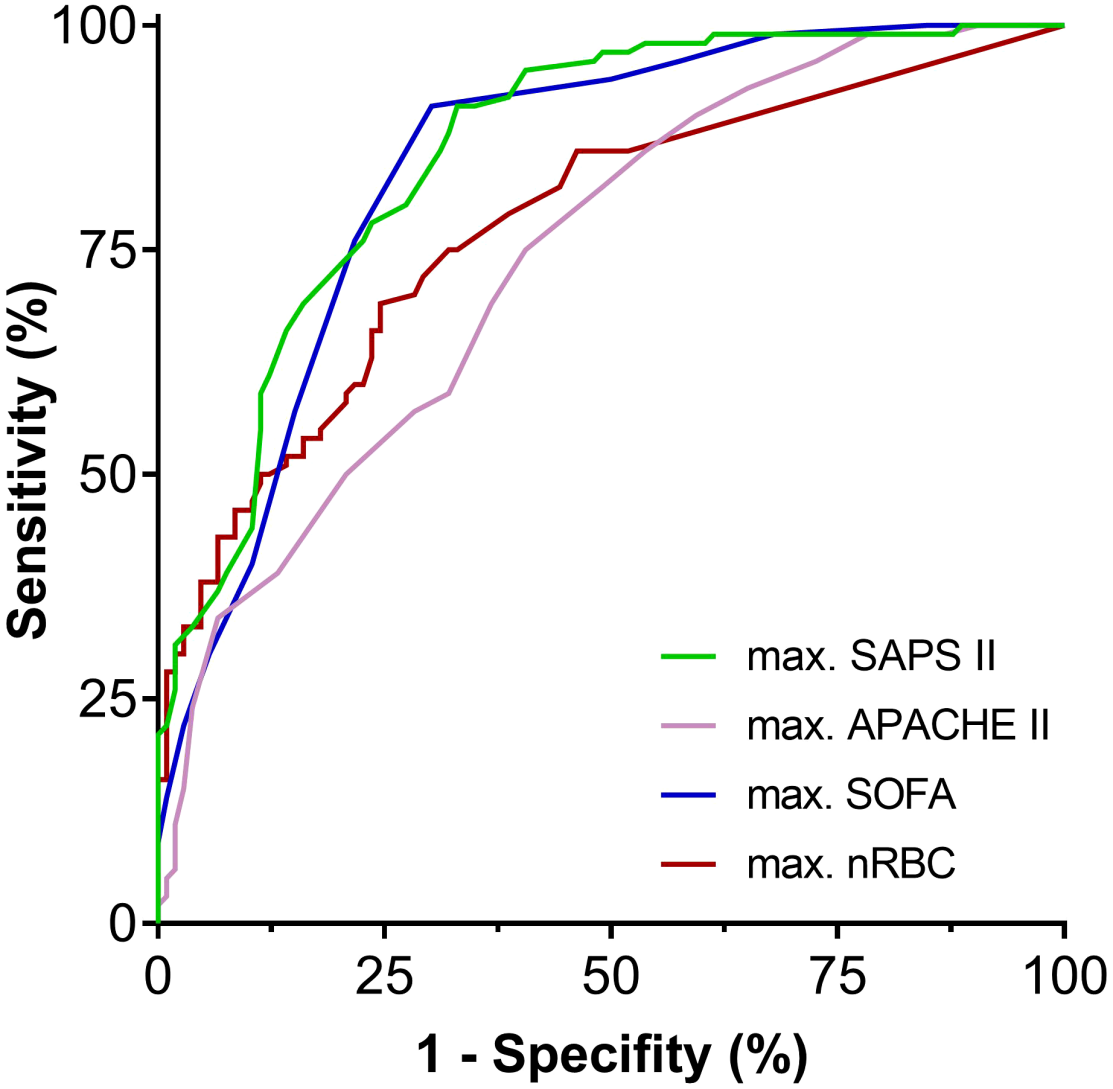
Receiver operating characteristic curves for the examined disease severity scores compared to the maximum nRBC count. APACHE II, Acute Physiology and Chronic Health Evaluation II; SAPS II, Simplified Acute Physiology Score II; SOFA, sepsis-related organ failure assessment score.

**Figure 7:**
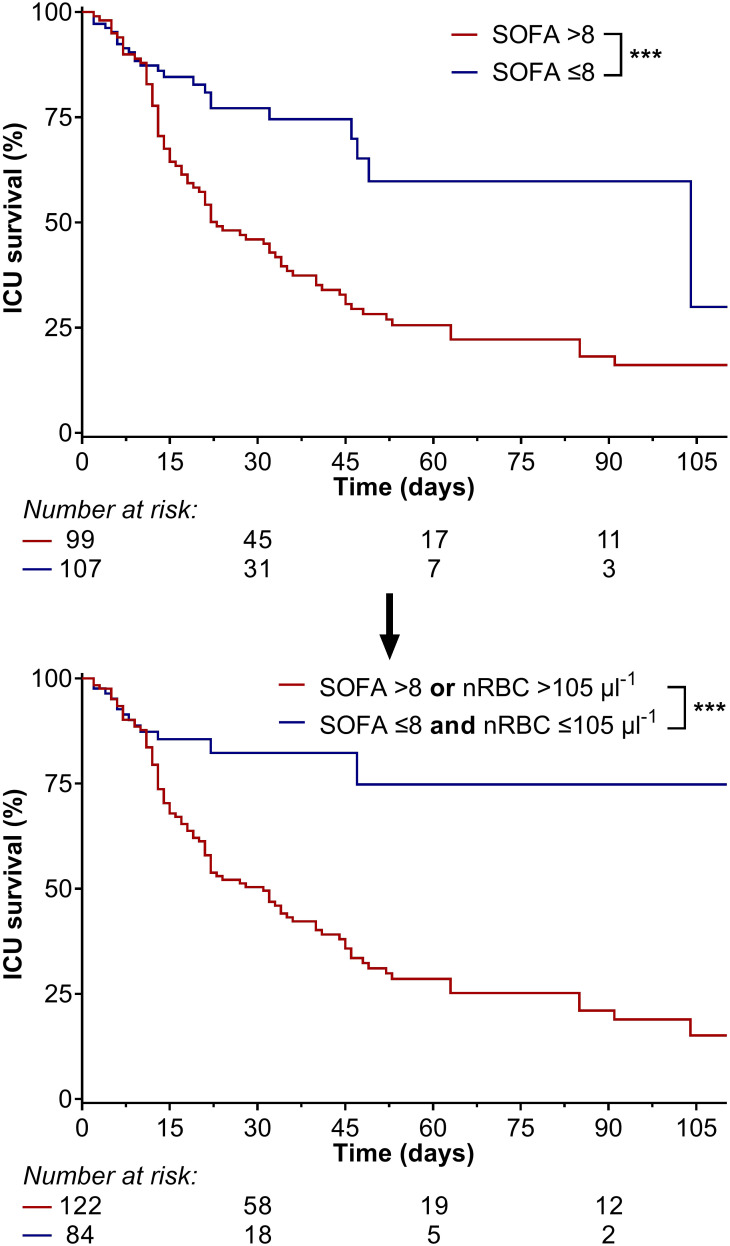
When the calculated maximal Sepsis-related Organ Failure Assessment (SOFA) score cut-off of >8 (survival rate: 16.1% vs. 29.9%) was combined with the maximum nRBC count cut-off of >105 µl^-1^, 23 patients were reclassified, and discrimination in intensive care unit survival rates considerably improved (maximal SOFA >8 or nRBC >105 µl^-1^: 15.1% vs. 74.8%). ***p < 0.001.

The receiver operating characteristic curves for maximum SOFA, APACHE II, and SAPS II scores compared to maximum nRBC counts are shown in **Figure 6**. The AUCROC for the maximum nRBC count was significantly smaller than those for the SOFA and SAPS II scores (SOFA: AUCROC 0.842, *p* < 0.001, cut-off 8, Sensitivity 91.0%, Specificity 69.8%, *p* = 0.038 vs. AUCROC max. nRBC; SAPS II: AUCROC 0.858, *p* < 0.001, cut-off 51, Sensitivity 91.0%, Specificity 67.0%; *p* = 0.017 vs. AUCROC max. nRBC) but did not differ significantly from that for the APACHE II score (AUCROC 0.740, *p* < 0.001, cut-off 25, Sensitivity 75.0%, Specificity 59.4%, *p* = 0.296 vs. AUCROC max. nRBC count). When the calculated maximal SOFA score cut-off of >8 (survival rates for maximal SOFA >8 16.1% vs. 29.9%, *p* < 0.001) was combined with the nRBC cut-off >105 µl^-1^, 23 patients were reclassified, and discrimination in ICU survival rates considerably improved (maximal SOFA >8 or nRBC >105 µl^-1^ 15.1% vs. 74.8%, *p* < 0.001; **Figure 7**).

### 3.6 Temporal connection of peak nRBC counts with disease severity scores and clinical parameters

**Figure 8:**
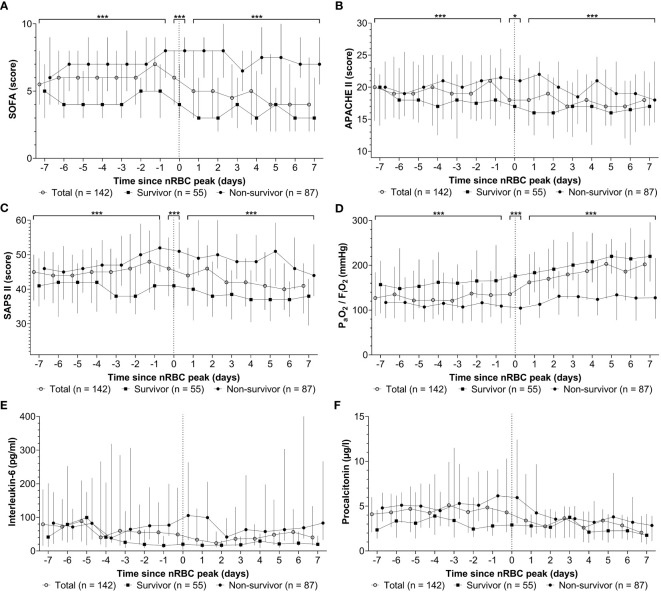
Among the patients with nucleated red blood cells (nRBC) positivity during their intensive care unit stay, the peak **(A)** sepsis-related organ failure assessment (SOFA) score, **(B)** Acute Physiology and Chronic Health Evaluation APACHE II score, **(C)** simplified acute physiology scores (SAPS) II score, **(D)** P_a_O_2_/F_i_O_2_, **(E)** interleukin-6 level, and **(F)** procalcitonin level occurred before the peak nRBC count. When the week before and after the peak nRBC count was examined, non-survivors had significantly higher SOFA, APACHE II, and SAPS II scores and lower P_a_O_2_/F_i_O_2_ values than survivors (brackets); no significant differences were observed in interleukin-6 and procalcitonin levels. *p < 0.05; ***p < 0.001.

The course of SOFA, APACHE II, and SAPS II, P_a_O_2_/F_i_O_2_, and IL-6 and PCT levels seven days before and after maximum nRBC count are shown in **Figure 8** and were compared between ICU survivors and non-survivors. Survivors had significantly lower SOFA, APACHE II, and SAPS II scores and significantly higher P_a_O_2_/F_i_O_2_ ratios before and after the peak nRBC count; IL-6 and PCT levels did not differ significantly between survivors and non-survivors. All peak SOFA, APACHE II, SAPS II, P_a_O_2_/F_i_O_2_, IL-6, and PCT values occurred significantly earlier than the peak nRBC count (**Table 4**). Furthermore, survivors reached their maximum SOFA, SAPS II, and IL-6 values and minimum P_a_O_2_/F_i_O_2_ values earlier than non-survivors. No differences were observed for nRBC, APACHE II, and PCT values.

**Table 4:** Intensive care unit treatment day on which maximum values nucleated red blood cell counts, disease severity scores, PaO2/FiO2 and inflammation parameters were observed.

	total (n = 142)	*p* (vs. day max. nRBC)	survivors (n = 55)	non-survivors (n = 87)	*p*
ICU day max. nRBC [IQR] - day	12 [7 - 21]		10 [6 - 20]	13 [7- 22]	0.158
ICU day max. SOFA [IQR] - day	10 [3 - 15]	<0.001	4 [1 - 14]	11 [6 - 16]	<0.001
ICU day max. APACHE II [IQR] - day	11 [4- 19]	0.034	12 [2- 19]	10 [4 - 19]	0.811
ICU day max. SAPS II [IQR] - day	9 [4 - 15]	0.002	5 [2 - 13]	11 [5 - 18]	0.002
ICU day min. P_a_O_2_/F_i_O_2_ [IQR] - day	5 [3 - 12]	<0.001	4 [2 - 7]	7 [3 - 16]	0.003
ICU day max. interleukin-6 [IQR] - day	7 [2 - 14]	<0.001	4 [2 - 9]	9 [3 - 15]	0.005
ICU day max. procalcitonin [IQR] - day	9 [2 - 18]	0.001	6 [2 - 18]	10 [2 - 18]	0.269

APACHE II, Acute Physiology and Chronic Health Evaluation II; ICU, intensive care unit; nRBC, nucleated red blood cells; SAPS II, Simplified Acute Physiology Score II; SOFA, sepsis-related organ failure assessment score.

## 4 Discussion

Our study demonstrated that the maximum nRBC count predicts disease severity and ICU mortality in patients with COVID-19 ARDS. A maximum nRBC count of >105 µl-1 had the best predictivity, and ICU mortality risk generally increased incrementally with the maximum nRBC count. Our data indicate that nRBC were observed due to aggravating disease severity and inflammation after the peaks in disease severity scores, PaO2/FiO2, and inflammatory markers.

In general, nRBC positivity has varied profoundly among critically ill patients in different study cohorts. In our study, 68.9% of patients tested positive for nRBC at least once during their ICU stay, which is generally consistent with previous data showing 75.5% nRBC positivity in a non-COVID-19-associated ARDS cohort (12). However, fewer positive nRBC measurements were seen in cardiac ICU patients (54.6%), critically ill surgical patients (27.6%), and medical ICU patients (17.5%) (10, 11, 25). In these cohorts, peak nRBC counts were also found to significantly predict ICU mortality. Therefore, our data on patients with severe COVID-19 are consistent with other critically ill patient cohorts. However, since nRBC positivity at ICU admission failed to significantly predict mortality, it is not applicable in stratifying for disease severity at ICU admission.

In patients with non-COVID-19 ARDS, peak nRBC counts were observed at day 7 in non-survivors and day 5 in survivors (12). However, in our COVID-19 ARDS cohort, peak nRBC counts were delayed at day 13 in non-survivors and day 10 in survivors, respectively. This difference is particularly striking because many patients were transferred to our ICU from a lower care level hospital where ICU treatment had already begun. Therefore, despite our other findings, a positive nRBC measurement at ICU admission did not improve risk stratification. Furthermore, outcome analysis of the nRBC count at pre-defined days might be insufficient based on the current evidence. However, a small cohort study comprising only 71 ICU patients with COVID-19 showed that the nRBC count on day 7 could be used to predict mortality (26). Nonetheless, our results showed that the nRBC count at admission cannot predict adverse outcomes despite Kaplan-Meier survival estimates being highly comparable within the first 15 days in the ICU.

While evidence has emerged that SARS-CoV-2 can affect bone marrow function and trigger subsequent leucoerythroblastic reaction in patients with COVID-19, the nRBC measurements did not seem to be a specific effect of SARS-CoV-2 in our study, given the available evidence from other clinical syndromes (20, 27). However, despite nRBC positivity, blast cells were found at least once in 22.8% of our study cohort. This finding might not only be attributed to overall disease severity since significantly more nRBC have been found in the peripheral blood of SARS-CoV-2 positive than negative emergency department patients (28).

Our analysis among nRBC-positive patients showed that ICU death is associated with a recent nRBC peak, with most deceased patients not surviving the first day after their individual nRBC peak. Since similar findings have been reported in patients with non-COVID-19-ARDS, high nRBC counts should be considered a clinical alarm signal (12).

Nevertheless, the clinical benefit of improving risk stratification through nRBC measurement might be impaired given our data showing that nRBC peaks were considerably delayed compared to the peaks in the examined disease severity scores and clinical parameters, such as P_a_O_2_/F_i_O_2_, IL-6, and PCT. Therefore, it must be questioned whether nRBC positivity might be the final but emerging result of severe hypoxemia and inflammation that only becomes apparent after other parameters have already indicated worsening critical illness (29). However, recent evidence has emerged that nRBC themselves might be immune cell mediators of the antiviral host response and can even provide immunosuppressive properties compromising the response against systemic infections (30, 31). These findings would align with the observation of the delayed occurrence of nRBC compared to other clinical measures in our study. However, jointly applying the nRBC cut-off with the SOFA score cut-off still greatly improved ICU mortality prediction in our cohort. Because our study - to our knowledge - is the first to analyze the time course of nRBC counts compared to other clinical parameters, it could be hypothesized that these findings might not only apply to patients with COVID-19 ARDS patients but also to other critically ill patient populations, which should be evaluated in further studies.

Furthermore, while nRBC measurements are widely available today and predictivity in distinct critically ill patient populations is well-known, no clinical recommendation exists for nRBC measurement in critically ill or ICU patients. While the distinct mechanisms leading to nRBC release remain unknown, it is negatively correlated with arterial oxygen saturation and, consequently, with hypoxemia, higher levels of erythropoietin and proinflammatory cytokines, such as IL-3 and IL-6 (16, 29, 32). Our study confirms these observations, showing lower P_a_O_2_/F_i_O_2_ and higher PCT values in patients with an nRBC count >105 µl^-1^. However, our study found no significant difference in IL-6 levels in daily or weekly intervals. Nevertheless, numerically higher values were observed in patients with an nRBC count >105 µl^-1^, and our statistical analysis might have been impaired by the relatively small numbers of measurements, which are broadly distributed.

When the time courses of clinical parameters were analyzed before and after the nRBC peak, non-survivors showed persistent inflammation, indicated by at least numerically persistent IL-6 levels, while IL-6 levels declined in survivors. Furthermore, P_a_O_2_/F_i_O_2_ did not improve in non-survivors in the days after the nRBC peak. Therefore, our study is consistent with other clinical data indicating that nRBC are found in the peripheral blood due to inflammation and hypoxemia and may not be an independent parameter.

While retrospectively combining clinical scores, such as the APACHE II and SOFA, with maximum nRBC count improved mortality prediction accuracy in critically ill patients, potential benefits for daily clinical practice remain questionable (25). Therefore, nRBC might be barely clinically applicable not only in assessing the mortality risk of patients with COVID-19 ARDS but potentially also of other critically ill patients. Nonetheless, nRBC positivity was still generally associated with post-discharge mortality in critical illness survivors (14). Therefore, nRBC positivity could trigger distinct post-discharge follow-up examinations counteracting these observations. However, further trials evaluating possible treatment strategies for post-discharge care would be needed.

Our study had several limitations. First, since it analyzed retrospective data, patient selection might have been influenced by data availability issues. However, nRBC measurements could not be indicated directly by the ICU physicians. Second, the analyzed patients were treated at a tertiary care level university hospital, and many had been transferred from lower care level hospitals where ICU treatment had already begun. Therefore, many of the analyzed patients had severe ARDS requiring invasive ventilation, prone positioning, and ECMO therapy. This finding might limit the generalizability of our findings because no data were available for the disease course before admission, including nRBC measurement. In addition, admission to the tertiary care hospital was performed at different time points, depending on the patients’ clinical conditions. Third, while nRBC measurement was a standard procedure within the blood count in our laboratory, not all patients received daily nRBC measurements in the ICU. Therefore, we had to include patients with at least one nRBC measurement every two days throughout their ICU stay. Third, the longer-term course of parameters shown in our analysis should be interpreted cautiously because fewer data might be available due to the high fraction of already deceased or discharged patients by those time points. Fourth, COVID-19 severity and clinical syndrome have changed since the start of the pandemic due to different SARS-CoV-2 variants, immunity, and vaccination status. Therefore, our data, mostly for patients with wild-type infection and no preexisting immunity, should be generalized cautiously. However, we found no evidence of different nRBC values among the different variants analyzed in our study.

In conclusion, our retrospective analysis showed that maximum nRBC counts predict ICU mortality and indicate disease severity among patients with COVID-19 ARDS. Compared to other established clinical scoring systems and laboratory parameters, the maximum nRBC count is a late predictor of ICU mortality. However, ICU mortality prediction was improved when combined with the SOFA score. The occurrence of high nRBC counts should be considered a clinical alarm signal. Further larger-scale studies should assess the connection between nRBC counts and other clinical parameters not only in COVID-19 ARDS but also in other critical illnesses where nRBC positivity has also been associated with adverse outcomes.

## Data availability statement

The raw data supporting the conclusions of this article will be made available by the authors, without undue reservation.

## Ethics statement

The studies involving humans were approved by ethics committee of the medical faculty of Justus Liebig University, Giessen, Germany (AZ 36/22). The studies were conducted in accordance with the local legislation and institutional requirements. The ethics committee/institutional review board waived the requirement of written informed consent for participation from the participants or the participants’ legal guardians/next of kin because data were anonymized and only derived from clinical and administrative records.

## Author contributions

GS: Conceptualization, Data curation, Formal analysis, Investigation, Methodology, Project administration, Resources, Supervision, Validation, Visualization, Writing – original draft, Writing – review & editing. AM: Conceptualization, Data curation, Formal analysis, Investigation, Methodology, Project administration, Writing – review & editing. CK: Conceptualization, Investigation, Methodology, Project administration, Resources, Supervision, Validation, Writing – review & editing. MM: Formal analysis, Investigation, Methodology, Project administration, Validation, Writing – review & editing. ES: Conceptualization, Data curation, Formal analysis, Investigation, Methodology, Project administration, Supervision, Validation, Writing – review & editing. UM: Conceptualization, Investigation, Methodology, Supervision, Validation, Writing – review & editing. MH: Conceptualization, Investigation, Methodology, Supervision, Validation, Writing – review & editing. KT: Conceptualization, Investigation, Methodology, Supervision, Validation, Writing – review & editing. MW: Conceptualization, Data curation, Investigation, Methodology, Project administration, Supervision, Validation, Writing – review & editing. MS: Conceptualization, Investigation, Methodology, Project administration, Resources, Supervision, Validation, Writing – review & editing. IV: Conceptualization, Formal analysis, Investigation, Methodology, Project administration, Resources, Supervision, Validation, Writing – review & editing.
